# Phenomic Selection for Hybrid Rapeseed Breeding

**DOI:** 10.34133/plantphenomics.0215

**Published:** 2024-07-24

**Authors:** Lennard Roscher-Ehrig, Sven E. Weber, Amine Abbadi, Milka Malenica, Stefan Abel, Reinhard Hemker, Rod J. Snowdon, Benjamin Wittkop, Andreas Stahl

**Affiliations:** ^1^ Department of Plant Breeding, Justus Liebig University, Giessen, Germany.; ^2^ NPZ Innovation GmbH, Holtsee, Germany.; ^3^ Limagrain GmbH, Peine-Rosenthal, Germany.; ^4^ Julius Kuehn Institute (JKI), Federal Research Centre for Cultivated Plants, Institute for Resistance Research and Stress Tolerance, Quedlinburg, Germany.

## Abstract

Phenomic selection is a recent approach suggested as a low-cost, high-throughput alternative to genomic selection. Instead of using genetic markers, it employs spectral data to predict complex traits using equivalent statistical models. Phenomic selection has been shown to outperform genomic selection when using spectral data that was obtained within the same generation as the traits that were predicted. However, for hybrid breeding, the key question is whether spectral data from parental genotypes can be used to effectively predict traits in the hybrid generation. Here, we aimed to evaluate the potential of phenomic selection for hybrid rapeseed breeding. We performed predictions for various traits in a structured population of 410 test hybrids, grown in multiple environments, using near-infrared spectroscopy data obtained from harvested seeds of both the hybrids and their parental lines with different linear and nonlinear models. We found that phenomic selection within the hybrid generation outperformed genomic selection for seed yield and plant height, even when spectral data was collected at single locations, while being less affected by population structure. Furthermore, we demonstrate that phenomic prediction across generations is feasible, and selecting hybrids based on spectral data obtained from parental genotypes is competitive with genomic selection. We conclude that phenomic selection is a promising approach for rapeseed breeding that can be easily implemented without any additional costs or efforts as near-infrared spectroscopy is routinely assessed in rapeseed breeding.

## Introduction

A key element of plant breeding is to select the best performing accessions with desirable traits. For this purpose, the breeding material traditionally needs to be phenotyped, which can be very laborious, time-consuming, and costly. The advent of genomic selection (GS) enabled breeders to make decisions about unphenotyped individuals based on predictions of genotypic values derived from their genotypic information [1–3]. GS utilizes a vast number of genetic markers, such as single-nucleotide polymorphisms (SNPs), to estimate genomic estimated breeding values (GEBV). The GEBV of an accession is based on the cumulative effect of each individual marker. To estimate these effects, the prediction model is trained on a set of individuals with both genotypic and phenotypic data, referred to as the training set. Trained models are able to predict the GEBV of unphenotyped accessions. Based on the predicted GEBV, the phenotypic values of the trait under consideration, i.e., the performance of the accession, can be calculated. This process can be referred to as genomic prediction (GP). However, both terms, GS and GP, are often used interchangeably. Breeders can use the predicted phenotypic values or GEBVs to identify promising candidates at very early developmental stages, or even directly from seeds. By performing predictions within the test set, which comprises individuals whose performance is both measured and predicted, the prediction accuracy of different models can be evaluated and compared. A commonly used model for GS is the genomic best linear unbiased prediction model (GBLUP). In this model, all available SNP markers are used to estimate a genomic relationship matrix between genotyped individuals [4,5]. The GEBVs are estimated based on this genomic relationship matrix, rather than on each individual marker, which reduces the dimensionality of the data used by the prediction model. With the ability to phenotype only the individuals of the training set, while relying solely on genotyping for the remaining individuals, GS enables the selection of a greater number of individuals within a shorter timeframe. As a result, the cycle length of a breeding program can be reduced, while the expected genetic gain increases [6,7]. The efficiency of genotyping technologies has remarkably increased over time, while the associated costs have decreased [8], allowing for the extensive identification of genetic markers distributed throughout the genomes of numerous distinct species. This has led to the adoption of GS as a routinely applied tool for breeding programs of many crops [9,10].

However, the extensive implementation of genotyping still faces obstacles in certain breeding programs where the cost of genotyping a substantial number of newly developed individuals per generation remains prohibitive [6]. Additionally, challenges arise when dealing with species lacking suitable resources for genotyping, such as SNP arrays. Consequently, there is a growing interest in alternative methods that can provide cost-effective and easily obtainable variables for efficient phenotype prediction.

Phenomic selection (PS), first described by Rincent et al. [11], has emerged as one such promising alternative. PS employs easily accessible phenotypic values, such as reflectance measured via near-infrared spectroscopy (NIRS), instead of genotypic data, to predict phenotypic traits like seed yield. While it has previously been shown that NIRS has a capability to predict such complex traits for individual samples within plots [12–16], the concept of PS extends this capability to the level of the genotypic value by using NIRS-derived data as regressors or to estimate kinship in a statistical model comparable to GBLUP. This enables the prediction of traits across different environments or experiments, which potentially allows the selection of favorable lines based on NIRS profiles collected in a specific reference environment, analogous to how SNP profiles are used in GS. By applying PS to winter wheat, Rincent et al. [11] achieved accuracies for the prediction of yield that surpassed those obtained by using SNP data in a GBLUP. Subsequent studies have validated the successful application of PS based on NIRS data in wheat [17–19] and other species such as maize [20,21], grapevine [22], triticale [23], and soybean [24].

PS holds several advantages over GS, most notably its applicability to any species with minimal sample preparation requirements, its rapidity, and its cost-effectiveness. Unlike GS, PS does not typically require prior treatment of samples, such as DNA extraction. NIRS can be directly applied to intact seed of many plants, enabling nondestructive measurements. Furthermore, the required equipment is substantially more affordable, making it accessible even for smaller laboratories and eliminating the need to outsource to external service providers. Technological advancements have even made it possible to collect spectral data at various growth stages and directly in the field, using portable spectrometers or unmanned aerial vehicles equipped with hyperspectral cameras. Recent studies have shown that such spectral data is suitable for PS as well [25–29]. Many breeding programs already routinely collect NIRS data for assessing the content or composition of specific plant metabolites. Consequently, the transition to PS would not necessitate additional costs associated with data collection, and the workload primarily involves analyzing the existing data.

In rapeseed breeding, NIRS is a well-established tool for evaluating seed quality traits, such as oil, protein, and glucosinolate contents, as well as the composition of fatty acids [30–32]. Hence, the integration of PS could substantially enhance and expedite breeding programs. A key challenge, however, arises from the prevalent use of hybrid varieties in rapeseed production. The previous studies on PS have primarily focused on utilizing NIRS data obtained within the same generation as the traits being predicted. This mainly limits the applicability to line breeding scenarios, where selected genotypes are propagated through self-crossing, thereby maintaining genetic stability across generations. However, for hybrid varieties the desired plants are the first filial (F_1_) generation resulting from crosses between 2 distinct homozygous parental lines. This causes a shift in the genotypic composition across generations. The genotype of a hybrid arises from the combination of the genotypes of its parental lines. Consequently, there is a need to investigate whether NIRS profiles of the parental lines can sufficiently capture the genotypic effects passed on to the next generation and how these effects impact the expression of the targeted trait in the hybrid.

To make use of the heterosis effect, breeders strive to maximize the genetic distinctness of the parents by forming complementary heterotic pools of germplasm [33–36]. Heterosis, also known as hybrid vigor, describes the effect that the F_1_ generation as offspring from genetically diverse, homozygous parents exhibits a higher phenotypic performance as both parents. The main goal is to identify the combination of 2 lines, each from one pool, that results in the best performing hybrid. This process is limited by the practicality of producing inbreds, to obtain homozygosity in the parental line, and testing all possible crosses. Therefore, hybrid breeding typically involves a 2-step approach where candidates from one heterotic pool are crossed with a few testers of the complementary pool to evaluate their general combining ability (GCA). Subsequently, selected lines of both groups are crossed in a factorial design to evaluate their specific combining abilities and to finally select the best combination for hybrid production [37,38]. Predicting the performance of potential crosses without actually carrying them out drastically increases the efficiency of hybrid breeding programs. As summarized by Seye et al. [38], many studies have applied GS models based on genotypic information of potential parents to predict hybrid performance in various crops, including rapeseed [39]. Therefore, to evaluate the applicability of PS to hybrid breeding, the key question is whether NIRS profiles from parental genotypes can be used to effectively predict traits in the resulting hybrids, i.e., across generations.

Our study aims to explore the feasibility and potential value of implementing PS in rapeseed breeding programs, specifically by applying it to a structured hybrid population and comparing its prediction accuracy with that of GS. We utilized NIRS profiles obtained from harvested seeds of both hybrid plants and parental plants to compare the ability to predict key traits, such as seed yield, flowering time, and plant height within and across generations. Besides using NIRS data collected in multiple environments, we tested the ability of NIRS profiles obtained from single locations to predict traits measured at multiple environments. Given that the examined population was derived from a structured crossing scheme, we furthermore implemented a familywise cross-validation methodology to investigate the influence of population structure on PS compared to GS. In addition to the originally proposed prediction model, we used various linear and machine learning approaches to compare their prediction accuracies.

## Materials and Methods

### Plant material and field trials

**Fig. 1.**
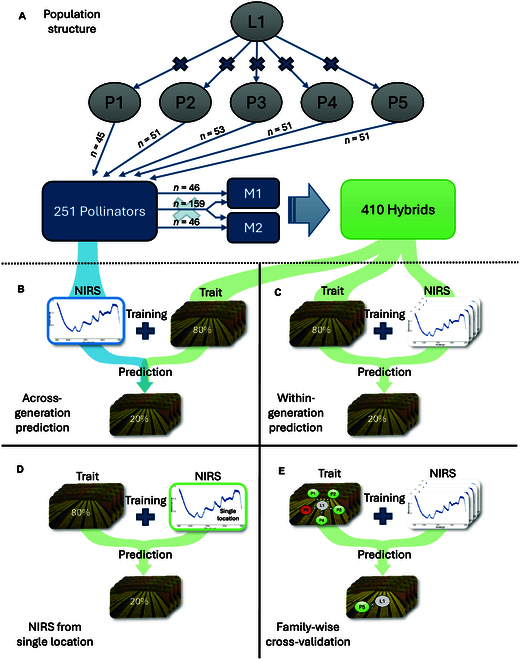
Overview of the experimental design. The rapeseed population used in this study was based on crossings of 5 different founder lines (P1 to P5) with a common elite line (L1). The resulting 251 pollinators were crossed with 2 different male-sterile inbred lines (M1 and M2), resulting in 410 test hybrids (A). Across-generation prediction was performed by using NIRS data obtained from the pollinators, grown at 1 location, to predict phenotypic traits of the hybrids, grown at 5 locations (B). Within-generation predictions were performed by using NIRS data and phenotypic traits both obtained from the hybrids (C to E). Here, phenotypic traits were obtained from all 5 locations, while NIRS data was obtained either from all 5 locations (C and E) or from single locations (D). Cross-validation was performed by randomly dividing the hybrid population into 80% for the training set and 20% for the test set with 200 repetitions (B to D) or by using hybrids, which descend from 4 of the 5 original crosses as the training set and the remaining subfamily as test set (E).

The winter oilseed rape population used in this study consisted of 410 test hybrids. The origin of this population is illustrated in Fig. 1A. These hybrids were created by crossing a set of 251 homozygous genotypes with 2 distinct testers (labeled M1 and M2, where “M” stands for “mother”). Two inbred lines exhibiting male sterility were selected as testers in order to prevent self-fertilization. Accordingly, for these crosses, the 2 testers served as the female parents (mothers), while the set of 251 genotypes were used as male parents, hereafter referred to as pollinators. The set of 251 pollinators was created by crossing 5 founder lines (labeled P1 to P5, where “P” stands for “parent”) with a common elite line (L1). This resulted in a population structure with 5 subfamilies, each comprising 45 to 53 different genotypes. Of these 251 pollinators, 159 were crossed with both testers, while 46 were crossed only with one of the testers and another 46 were crossed only with the other tester (Fig. 1A). All crossings were carried out by the commercial breeders NPZ Innovation GmbH (Holtsee, Germany) and Limagrain GmbH (Peine-Rosenthal, Germany).

The field trials were performed in the seasons 2019/2020 (2020) and 2020/2021 (2021) at 5 different locations across Germany, namely: Rauischholzhausen (RHH), Hohenlieth (HOH), Moosburg (MOO), Rosenthal (ROS), and Lauenau (LAU). The field design was a partially replicated design with 1.2 replicates; that is, 20% of the genotypes within each environment were replicated once, while the remaining 80% were unreplicated. Replicates were chosen so that each genotype was repeated once in total across all locations. In addition to the test hybrids, 3 different check varieties were grown with 10 replications in each environment. In the context of this study, these check varieties were used for a more precise adjustment for field design effects. In both years, the 251 pollinators were also grown at one of the locations (RHH). The check varieties were again included with 10 replicates each, while the pollinators themselves were not replicated.

### Phenotypic data

The traits assessed in this study, derived from the hybrids, included seed yield, plant height, flowering time, as well as the oil and protein content of the harvested seeds. Plant height was determined after flowering as the average of all plants within a plot. Flowering time was defined as the start of flowering in days from January 1. Oil and protein content were determined using NIRS and, like the seed yield, were adjusted to a standard water content of 9%. In one environment (LAU 2020), values of 188 plots were excluded from further analysis due to waterlogging. Additionally, plant height was not measured in LAU, and both plant height and flowering time were not recorded in RHH in 2020. With the remaining data, adjusted entry means for each trait were calculated using the following linear mixed model:

$$\begin{array}{c} y_{\text{ijklmn}}=\mu +g_{i}+e_{j}+r_{k(e_{j})}+c_{l(e_{j})}+a_{m}+l_{n}+(g_{i}\times e_{j})+\varepsilon \end{array}$$ (1)

where *y_ijklmn_* represents the observed phenotypic data for each plot. The overall mean *μ* and the effect of the *i*th genotype *g_i_* are defined as fixed factors, whereas the remaining effects are defined as random factors. The effect of the *j*th environment is included as *e_j_*, while *r*_*k*(*e_j_*)_ and *c*_*l*(*e_j_*)_ represent the effects of the *k*th row and *l*th column within the *j*th environment, respectively. Furthermore, *a_m_* stands for the effect of the *m*th year, *l_n_* for the effect of the *n*th location, (*g_i_* × *e_j_*) for the genotype-by-environment interaction effect between the *i*th genotype and the *j*th environment, and *ε* represents the residual error. Subsequently, the adjusted mean for each genotype was obtained as *μ* + *g_i_*.

The heritability of each trait, meaning the proportion of variation in this trait that can be attributed to inherited genetic factors, was estimated using an approach for unbalanced data [40,41] as:

$${\bar{H}}^{2}=\frac{\sigma_{g}^{2}}{\sigma_{g}^{2}+{\bar{v}}_{∆}^{\text{BLUE}}/2}$$ (2)

where $\sigma_{g}^{2}$ is the genotypic variance, obtained from a model as described above but considering genotype as random effect. ${\bar{v}}_{\text{∆}}^{\text{BLUE}}$ is the mean variance of a difference between 2 adjusted genotype means (BLUEs), i.e., mean variance of all pairwise genotype contrasts.

### NIRS data

NIRS was performed on the harvested seeds of the pollinators grown in RHH and hybrids grown at all 5 locations in both years, using a SpectraStar 2500X RTW (KPM Analytics, Westborough, MA, USA). Each sample consisted of 5 g of whole seeds bulked from a single plot. Each plot was measured using 2 technical replicates. The 188 plots in LAU, where waterlogging occurred in 2020, have been excluded. Reflectance was measured at each wavelength between 680 and 2,500 nm, resulting in a profile with 1,820 values for each plot. After averaging the technical repetitions, each reflectance value within the NIRS profiles of each hybrid was adjusted using the same linear mixed model applied to the phenotypic data, described in Phenotypic data. Again, *μ* + *g_i_* was used as adjusted value for each respective genotype. This adjustment process aggregated the multiple NIRS profiles of each genotype—measured across different environments and their respective replicates within those environments—into a single, adjusted NIRS profile per genotype. Essentially, NIRS profiles from multiple plots containing the same genotype were combined into one aggregated NIRS profile for each genotype. This improves the ability of the NIRS profiles to serve for the prediction of genotypic values, as described by Robert et al. [42]. For the parental NIRS profiles, as well as for a prediction scenario where only the hybrid NIRS profiles obtained from single locations were used to predict the performance across all locations, the model was reduced by the location and environment term:

$$y_{\text{iklm}}=\mu +g_{i}+a_{m}+r_{k\left(a_{m}\right)}+c_{l\left(a_{m}\right)}+\varepsilon$$ (3)

Afterwards, to correct for technical effects occurring during the measurement, which influence the measured reflectance independently of the specific composition of the seeds, a mathematical correction was applied to the adjusted NIRS profiles, as described by Robert et al. [42]. These technical effects typically occur as baseline shift, where measured reflectance values increase at higher wavelengths due to the increased light density. This artificial trend can be corrected by utilizing the derivative of the NIRS profile, which enhances the true features of the spectrum. Therefore, the first derivative of each adjusted NIRS profile was calculated using a Savitzky–Golay algorithm with a window size of 37 data points, implemented in the R package “prospectr” [43]. This resulted in profiles with 1,785 wavelengths ranging from 698 to 2,483 nm, which were then centered and scaled. These preprocessed NIRS profiles then served as an input for the calculation of a NIRS-based relationship matrix or directly for the different prediction models as described later in Prediction models. For 9 pollinators, no NIRS data was available.

### Genotypic data

Genotypic data was obtained from the pollinators using the Brassica 15K Illumina Infinium SNP array (SGS-TraitGenetics GmbH, Gatersleben, Germany), which consists of a subset of the 60K Illumina Infinium SNP array [44]. All SNP markers were filtered for single-copy BLAST hits of their flanking sequences to remove markers with nonunique positions on the Brassica napus Express 617 reference genome v2 [45]. After discarding markers that had more than 2 alleles and more than 10% missing values, and filtering for an expected heterozygosity of ≥0.095, the number of remaining markers used for GPs was 6,200. For 6 pollinators, no genotypic data was available. Additionally, 2 SNP profiles had to be excluded since a principal component analysis (PCA) indicated a labeling error. Hence, 399 test hybrids were used for the predictions since both genotypic and NIRS data were available.

### Population structure

The population structure of the hybrid set was assessed by conducting a PCA based on SNP markers, as well as on adjusted NIRS profiles, which were obtained from the pollinators. The first 3 principal components were used to visualize the population structure.

### Prediction models

Five different statistical models were compared, considering their ability to predict hybrid performance using either SNP profiles, adjusted NIRS profiles, or a combination of both as predictor. According to Lane et al. [20], we decided to denote the model that is equivalent to the GBLUP as NIRS-BLUP. To cover both parametric and nonparametric models, 2 Bayesian methods and 2 machine learning methods were used additionally. The Bayesian models tested are the Bayesian LASSO (BL) model, which has the ability of marker-specific shrinkage, and the semiparametric reproducing kernel Hilbert space (RKHS) regression, allowing the modeling of higher-order epistasis. The machine learning algorithms are random forest (RF) and support vector machines (SVMs).

The linear and Bayesian models are explained below in the full form, which was used when the combination of both SNP and NIRS profiles were used as predictors. The corresponding terms for the marker and NIRS effects were omitted or included, depending on the inclusion of the respective effect in the model.

In the GBLUP/NIRS-BLUP, the underlying linear mixed model is:

$$y=\mathrm{X\beta}+Z_{a}a+Z_{\text{NIRS}}i+e$$ (4)

where *y* is a vector of observations for the trait under consideration. *X* is a design matrix relating fixed effects *β* to the phenotypic records and consists out of a column for the intercept and a column with a one-hot encoded variable for the male-sterile mother of the hybrid. *Z_a_* and *Z_NIRS_* are the design matrices for the random effects, *a* is a vector of random additive marker effects, *i* is a vector of random effects due to the adjusted NIRS profiles, and *e* is the random residual term. It is assumed that *a*, *i*, and *e* follow a normal distribution, accordingly:

$$\begin{array}{c} a\;∼\;N\left(0,G_{a}\sigma_{a}^{2}\right),\;i\;∼\;N\left(0,G_{\text{NIRS}}\sigma_{i}^{2}\right),\text{and}\;e\;∼\;N\left(0,I\sigma_{e}^{2}\right) \end{array}$$ (5)

with $\sigma_{a}^{2}$, $\sigma_{i}^{2}$, and $\sigma_{e}^{2}$ being the additive genetic variance, the variance due to the NIRS profiles, and the error variance, respectively. *G_a_* is the genomic additive relationship matrix, *G_NIRS_* is the relationship matrix based on NIRS profiles, and *I* is an identity matrix. The additive genomic relationship matrix was calculated following VanRaden [5]:

$$G_{a}=\frac{{\mathrm{ZZ}}^{\prime }}{2\sum p_{i}\left(1-p_{i}\right)}$$ (6)

the elements of *Z* being (0-2p_i_) for homozygous allele A, (1-2p_i_) for heterozygous, and (2-2p_i_) for homozygous allele B, with *p_i_* being the allele frequency of the B allele. *Z* has the dimensions *n* × *m*, with *n* being the number of genotypes and *m* being the number of markers. The NIRS-based relationship matrix was calculated as assumed by Rincent et al. [11]:

$$G_{\text{NIRS}}=\frac{{\mathrm{WW}}^{\prime }}{l}$$ (7)

where *W* is the matrix containing the preprocessed NIRS profiles with dimensions *n* × *l*. *n* is the number of genotypes and *l* is the number of wavelengths. The linear mixed models were implemented and solved with the R package “sommer” [46,47].

The formula describing the BL model, following Park and Casella [48], is:

y=Xβ+Ma+Wi+e (8)

Here, *y* is the vector of observations for the trait under consideration, *X* is the design matrix as described above for the GBLUP/NIRS-BLUP, and *β* is the corresponding vector of fixed nongenetic effects. *M* is an incidence matrix with the marker profile with entrances 0 for homozygous allele A, 1 for heterozygous, and 2 for homozygous allele B; *a* is a vector of genetic additive effects, *W* is the matrix of the preprocessed NIRS profiles, and *i* is a vector of their effects. The coefficients of the fixed effects (*β*) are assigned flat priors, and the coefficients of the marker effects (*a*) and NIRS effects (*i*) are assigned double-exponential priors. This allows the shrinkage of some marker/spectra effects to effectively zero, introducing sparsity into the model. *e* represents the random residual term. These models were conducted in the R software with the package “BGLR” [49] with default parameters.

According to de los Campos et al. [50], the RKHS model with kernel averaging has the following form:

$$y=X\beta +\sum_{l=1}^{L}u_{l}+\sum_{l=1}^{L}i_{l}+e$$ (9)

with

$$\begin{array}{c} p\left(\beta ,u_{1},\ldots u_{L},i_{1},\ldots i_{L},e\right)\propto \prod_{l=1}^{L}N\left(u|0,K_{\mathrm{ul}}\sigma_{\mathrm{ul}}^{2}\right),\\ \prod_{l=1}^{L}N\left(i|0,K_{\text{NIRS}\;l}\sigma_{\text{NIRS}\;l}^{2}\right),N\left(e|0,I\sigma_{e}^{2}\right) \end{array}$$ (10)

Here, *y* is the vector of observations, while *K_ul_* and *K_NIRS l_* are *n* × *n* kernel matrices calculated based on the Euclidean distance between genotypes based on SNPs and NIR spectra, respectively. The kernel matrices were calculated with the Gaussian kernel with the *l*th value of the bandwidth parameter {0.1, 0.5, 2.5}. *Xβ* is treated similar as in the BL, and *u_l_* and *i_l_* are assumed to be random. In this way, the different random effects, i.e., the 3 kernel matrices from the 3 bandwidth parameters, are weighted by their variance components. The random residual term is *e*. This model was conducted in the R software with the package “BGLR” [49] with default parameters.

For the machine learning methods, hyperparameters were optimized with a Bayesian-model-based optimization approach. For this purpose, an objective function was defined first, which aims to minimize the out-of-bag error for RF or root mean squared error for SVM, respectively, by choosing hyperparameters within a specified range. These functions were implemented using the R package “smoof” [51]. After testing an initial design with 12 steps, where the hyperparameters are randomly chosen within the specified range, the objective function is optimized sequentially through gaussian process smoothing with 6 iterations using the R package “mlrMBO” [52]. The parameter space in which the approach is seeking for the optimal hyperparameter combination was defined using the R package “ParamHelpers” [53].

In RF, the prediction is based on the average outcome of an ensemble of multiple decision trees, where each tree uses a bootstrapped sample of the training set. It was first proposed by Breiman [54], and its application for GP is described in detail by González-Recio and Forni [55]. The underlying formula is:

$$y=\mu +\sum_{t=1}^{T}c_{t}h_{t}\left(y;X\right)$$ (11)

where *y* is the vector of observations, *μ* is the overall mean, *T* is the number of built decision trees, *c_t_* is a shrinkage factor that controls the contribution of each tree to the prediction, and *h_t_*(*y*; *X*) is the function describing the decision trees. Here, *X* is a centered and scaled matrix containing the SNP profiles, preprocessed NIRS profiles, or the combination of both. Additionally, a column for the male-sterile mother of the hybrids was added. This model was implemented with the R package “ranger” [56]. Hyperparameter “mtry” was optimized within a range between 100 and one-third of the number of columns of the respective input matrix *X*. For the optimization of “min.node.size” the range was defined between 3 and 15. The number of trees was set to 500.

The SVM algorithm implemented here is the ε-support vector regression [57]. It performs regression by projecting the data into higher dimensional space with a kernel function. For that, the r package “kernlab” [58] was utilized and the Radial Basis Function was used as kernel function. The spaces for hyperparameter optimization were defined as 0 to 2^10 for “C” and 0 to 0.5 for “epsilon”.

### Different prediction scenarios and evaluation of prediction accuracy

Phenomic prediction (PP) was evaluated in various scenarios, each differing based on the origin of NIRS data used for training the models. A key distinction is made between predictions within generation and across generation. For within-generation predictions, both NIRS data and the predicted phenotypic traits were obtained from the hybrids themselves (Fig. 1C to E). This approach was further subdivided into a scenario utilizing NIRS profiles adjusted across all environments (Fig. 1C) and a scenario where the NIRS data was collected from single locations (Fig. 1D). In contrast, across-generation predictions used NIRS profiles from the parental generation, specifically the pollinators, to predict traits in the hybrid generation (Fig. 1B). In both instances, the traits predicted are adjusted across all environments.

The prediction accuracy was evaluated through cross-validation, conducted in 200 runs. In each run, the population was randomly divided into 80% of the hybrids as training set and the remaining 20% as test set. Each model was trained on the training set, and then the trained models were applied to predict the performance of the test set, which had masked phenotypic data. For the machine learning models, hyperparameter tuning was carried out as described in Prediction models on the training set in each run. The best parameters obtained from the tuning process were then used for training of the respective model. The accuracy of the predictions was assessed by calculating the Pearson correlation coefficient (*r*) between the adjusted means of the observed phenotypic values and the predicted phenotypic values of the test set.

Furthermore, to examine the impact of population structure on prediction accuracy, a familywise cross-validation scheme was employed (Fig. 1E). For that purpose, data from 4 out of the 5 subfamilies were used as the training set, while the remaining family served as the test set, for which the phenotypic data was masked and predicted using the trained models. This was repeated until each family served as test set once. Prediction accuracy was measured as described in the random cross-validation.

### Evaluation of selection accuracy

To evaluate the efficacy of PS in rapeseed breeding, an additional approach was performed to compare the selection accuracy of PS to GS and to the combined approach using both NIRS and SNP data. The selection accuracy was assessed by calculating the Czekanowski coefficient (CZ) of similarity between the selection of the best genotypes based on the adjusted means of the observed phenotypic values, i.e., the actual performance, and based on the predicted performance (averages from all cross-validations) for 2 different selection intensities. For this purpose, the best 80 genotypes and the best 40 genotypes, which is approximately 20% and 10% of the test hybrid population, were selected depending on the trait under consideration. Based on these selections, the genotypes were then categorized into 4 groups. A genotype that was selected both based on its predicted and its actual performance was classified as “correctly selected”. Accordingly, genotypes that were below the respective selection threshold by both predicted and actual performance were considered “correctly discarded”. Additionally, a genotype was tagged as “wrongly selected” if it was selected based on its predicted but not based on its actual performance. Similarly, a genotype was labeled as “wrongly discarded” if it was excluded based on predictions but actually belonged in the top 80 or top 40. Subsequently, CZ was calculated according to Qiao et al. [59] using the following formula:

$$\mathrm{CZ}=\frac{2a}{2a+b+c}$$ (12)

where *a* is the number of correctly selected genotypes, *b* is the number of wrongly selected genotypes, and *c* is the number of wrongly discarded genotypes. The coefficient serves as an objective indicator of selection accuracy, where a high value—close to 1—signifies that the selection made based on the predicted hybrid performance closely aligns with a selection based on the actual performance, and conversely, a lower value indicates a greater divergence between the selections.

### Software

All analyses were performed using R v4.1.2 [60]. Besides the above-mentioned packages, we used “ggplot2” [61], “ggfortify” [62], and “ggpubr” [63] for data visualization. In addition, the AI language model ChatGPT, developed by OpenAI, was used to refine the manuscript in terms of language and grammar.

## Results

### Predictions within the hybrid generation

**Fig. 2.**
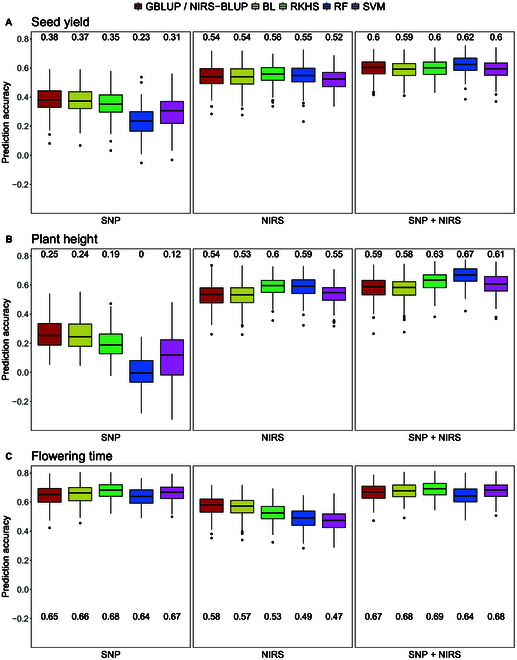
Comparison of prediction accuracy of GP based on SNP markers, PP based on NIRS data, and a combined approach based on both kinds of data with 5 different models (GBLUP/NIRS-BLUP, BL, RKHS, RF, and SVM) for seed yield (A), plant height (B), and flowering time (C) obtained by 200 cross-validation splits. Values above/underneath the boxplots represent median accuracies across all cross-validation runs. NIRS data was obtained within the hybrid generation from harvested seeds.

In the first prediction scenario, NIRS profiles obtained from the harvested seeds of the test hybrids were adjusted across all environments and used to perform within-generation predictions of the likewise adjusted traits seed yield, plant height, and flowering time (Fig. 1C). Statistical characteristics and heritability of each trait are listed in Table S1. PP was compared to GP and an approach based on a combination of both NIRS and SNP data (Fig. 2).

PP far outperformed GP for seed yield (Fig. 2A) and plant height (Fig. 2B) on each of the 5 models used. For seed yield, the median prediction accuracies achieved with PP ranged from 0.52 (SVM) to 0.56 (RKHS), with the base model NIRS-BLUP falling in the middle with a value of 0.54. In GP, on the other hand, the equivalent base model GBLUP achieved the highest accuracy of 0.38, and RF has the lowest value (0.23). For plant height, there was an even greater difference between PP and GP. Here, the median accuracies of the models using NIRS data range from 0.53 (BL) to 0.6 (RKHS), while the SNP-based models achieved values between 0.00 (RF) and 0.26 (GBLUP). In contrast, GP of flowering time (Fig. 2C) resulted in a high level of accuracy, which was not surpassed by PP. While the median accuracies for GP fell between 0.64 (RF) and 0.68 (RKHS), the highest accuracy achieved by using NIRS profiles was 0.58 (NIRS-BLUP), and the lowest value was 0.47 (SVM). Interestingly, the combined approach using both SNP and NIRS data resulted in the highest prediction accuracies for all 3 traits and with all 5 models. The best median value in this approach was 0.69, obtained by the prediction of flowering time using RKHS.

When comparing the accuracy of the different models, parallels between seed yield and plant height are noticeable. For both traits, the 5 models led to more similar results when NIRS profiles were used as predictors, while the results differed more when the models were based only on the genomic markers. In that case, the accuracy of the machine learning models RF and SVM was considerably below that of the other models. Remarkably, RF seemed to benefit the most from the combination of genomic and phenomic data as predictors. While the model performs worst in the SNP-based prediction of seed yield and plant height, it achieved the highest accuracies in the combined prediction approach. However, these patterns did not appear when predicting the flowering time. Here, the results of the individual models were closer to each other in GP than in PP. In addition, the accuracy of RF in the combined approach, in contrast to all other models, was not increased but remained at the same level as in GP. This shows that the prediction accuracy of the tested models varies greatly both between the traits and between the different predictors and that there is no model that generally performs best.

**Fig. 3.**
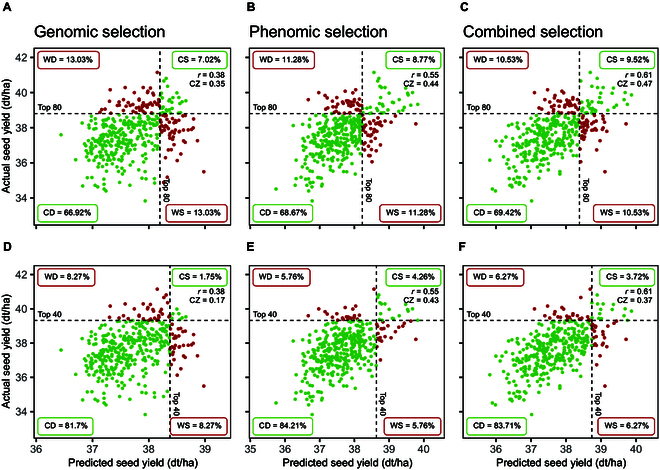
Comparison of selection accuracy for selecting the top 80 genotypes (A to C) or top 40 genotypes (D to F) with GS (A and D), PS (B and E), and combined selection (C and F) for seed yield based on the measured values (adjusted means) and predicted values (means of 200 cross-validations) with GBLUP/NIRS-BLUP. Genotypes that were in or out of the selected fraction based on both predicted and actual performance were classified as “correctly selected” (CS) or “correctly discarded” (CD), respectively. Genotypes that were in the selected fraction based on the prediction when in reality they performed worse, and vice versa, were classified as “wrongly selected” (WS) or “wrongly discarded” (WD), respectively. The respective percentage numbers indicate the corresponding faction sizes. Czekanowski coefficient of similarity (CZ) indicates the selection accuracy based on the predicted values. Pearson correlation coefficient (*r*) indicates the respective prediction accuracy. NIRS data was obtained within the hybrid generation from harvested seeds.

When using predicted phenotypic values to select the top 80 genotypes, the varying prediction accuracies were reflected in the selection accuracy. Figure 3 shows the accuracy of selecting for seed yield based on predictions with GBLUP/NIRS-BLUP as an example. Tables S2 and S3 list the results for all other traits and models. When selecting the top 80 genotypes GS correctly selected 7.02% of genotypes, which corresponds to 28 genotypes, while 13.03% (52 genotypes) were wrongly discarded or wrongly selected, respectively. This led to a CZ of 0.35 (Fig. 3A). PS achieved a correct selection of 8.77% (35 genotypes), and 11.28% (45 genotypes) were classified as wrongly discarded or wrongly selected, respectively, which resulted in a CZ of 0.44 (Fig. 3B). The combined approach outperformed both other approaches with a selection accuracy of 0.47 (Fig. 3C). In the selection of the top 40 genotypes, PS surpassed both GS and the combined approach gaining an accuracy of 0.43 (Fig. 3E). This trend was consistent across other prediction models as well (Tables S2 and S3).

For plant height selection, PS and the combined approach performed at comparable levels and generally better than GS, with minor variations depending on the model used. Corresponding to prediction accuracies, GS showed higher selection accuracies for flowering time when selecting the top 80 genotypes. However, in selecting the top 40 genotypes, GS was marginally outperformed by the combined approach.

### Predictions with NIRS data obtained from single locations

In a second prediction scenario, the hybrid NIRS profiles obtained from individual locations were used to predict the traits, which in turn were adjusted across all locations (Fig. 1D). For each of the 5 locations NIRS profiles were adjusted for effects of the year and field design. The objective was to evaluate whether a single reference location is sufficient to obtain NIRS profiles that are suitable for a representative prediction of the traits under consideration.

**Fig. 4.**
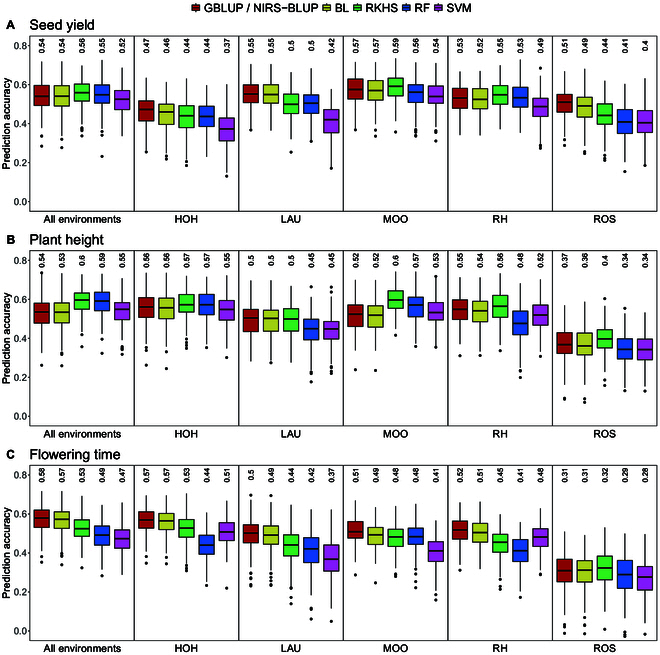
Comparison of prediction accuracy of PP based on NIRS data aggregated across all locations and obtained from single locations (HOH, LAU, MOO, RHH, or ROS) with 5 different models (GBLUP/NIRS-BLUP, BL, RKHS, RF, and SVM) for seed yield (A), plant height (B), and flowering time (C) obtained by 200 cross-validation splits. Values above the boxplots represent median accuracies across all cross-validation runs. NIRS data was obtained within the hybrid generation from harvested seeds.

In general, the obtained prediction accuracies were on a comparable level to the prediction scenario using aggregated NIRS profiles obtained from all environments (Fig. 4). When compared to the results of the GP shown in Predictions within the hybrid generation (Fig. 2), it can be observed that, regardless of the source location of the NIRS profiles used as predictors, each of the evaluated models demonstrated superior accuracy compared to their genomic counterparts in predicting seed yield and plant height. Concurrently, when it comes to predicting flowering time, GP still outperformed PP.

Comparing the accuracies across locations, it is worth noting that some locations consistently yielded higher accuracies, while others consistently had lower accuracies across all tested prediction models and traits (Fig. 4). NIRS profiles obtained from MOO and RHH always resulted in relatively higher accuracies. In relation to this, the predictions based on NIRS data from LAU achieved moderate accuracies, while predictions from ROS consistently had lower accuracies across all. Remarkably, HOH showed the lowest prediction accuracies for seed yield, whereas it achieved the highest median values for plant height and flowering time, respectively.

### Predictions of single subfamilies

**Fig. 5.**
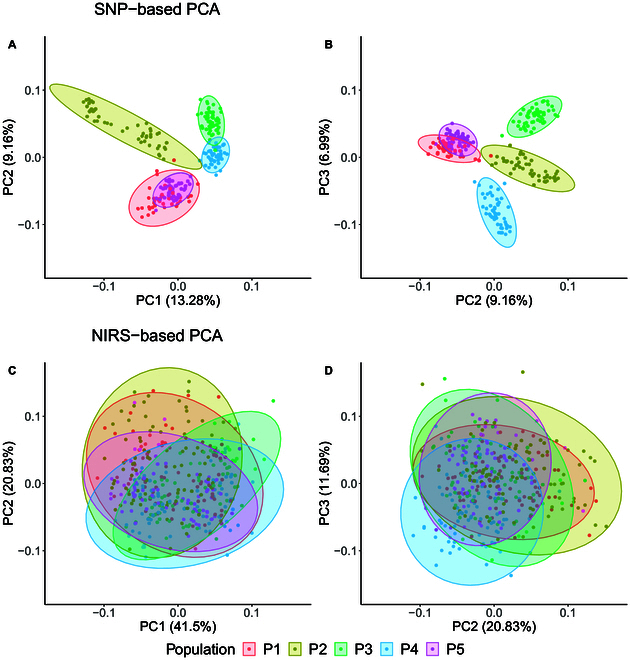
Population structure displayed by the first 2 principal components (A and C) as well as by the second and third principal components (B and D) based on SNP marker (A and B) and NIRS data (C and D). The colors indicate the affiliation to the 5 different subfamilies based on descent from the 5 founder lines. Both SNP and NIRS data were obtained from the pollinators.

A PCA using SNP profiles of the pollinators revealed distinct separation of 3 out of the 5 subfamilies, while the remaining 2 subfamilies overlapped when considering the first 3 principal components (Fig. 5A and B). In contrast, a notable overlap among all 5 subfamilies, with only a few individuals deviating from the main cluster, was observed when conducting a PCA based on NIRS profiles (Fig. 5C and D).

**Fig. 6.**
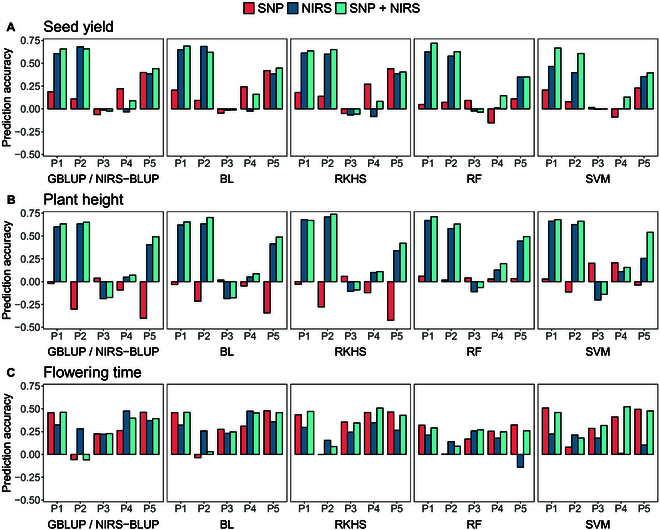
Comparison of prediction accuracy of GP based on SNP markers, PP based on NIRS data, and a combined approach based on both kinds of data for predicting the performance of one subfamily (P1 to P5) when trained on the remaining 4 subfamilies with 5 different models (GBLUP/NIRS-BLUP, BL, RKHS, RF, and SVM) for seed yield (A), plant height (B), and flowering time (C). NIRS data was obtained within the hybrid generation from harvested seeds.

The predictions in a familywise cross-validation scheme (Fig. 1E) unveiled variations in the accuracy of predicting the performance of distinct subfamilies, primarily influenced by predictors, target traits, and partially by prediction models. Figure 6 depicts the different prediction accuracies, while the exact values and median scores per model and trait can be found in Table S4.

For both seed yield (Fig. 6A) and plant height (Fig. 6B), PP and the combined approach often outperformed GP, especially when the test set comprised individuals derived from founder lines P1 and P2. Similar to random cross-validation within the whole population, the combined approach generally outperformed the exclusive utilization of NIRS data. However, the disparities were mostly marginal. In contrast to the predictions based on NIRS data, GP showed higher variation across models, with machine learning models differing from others and performed generally better for seed yield than for plant height. Flowering time predictions (Fig. 6C) showed less variation across families and predictors. Here, accuracies of the combined approach often matched levels of GP, whereas predictions based only on NIRS performed worse.

Upon direct comparison, the median prediction accuracies achieved through familywise cross-validation generally tended to be lower than those obtained from random cross-validation across the entire population (Fig. S1). These median disparities remained relatively consistent across predictors and models in seed yield predictions. However, for plant height predictions, the familywise cross-validated median accuracy aligned more closely with the median of random cross-validated predictions when NIRS or combined data were used with NIRS-BLUP or BL, compared to when these models were solely based on SNP profiles. Also, it appears more often that predictions for some subfamilies achieve accuracies surpassing the median of random cross-validation when NIRS and combined data were used. In the case of flowering time, the familywise cross-validated predictions exhibited the highest disparities to the random cross-validated predictions, in comparison to the other traits. Across all models and predictors, none of the subfamilies achieved a prediction accuracy surpassing the median of the random cross-validation approach. In certain instances, the highest value from familywise cross-validation was even lower than the lowest value from random cross-validation.

### Predictions across generations using parental NIRS profiles

To investigate the potential of predicting hybrid performance using NIRS data obtained in a prior generation, NIRS profiles of the 251 pollinators cultivated in RHH were employed as predictors for seed yield, plant height, flowering time, as well as oil and protein content of the hybrids.

**Fig. 7.**
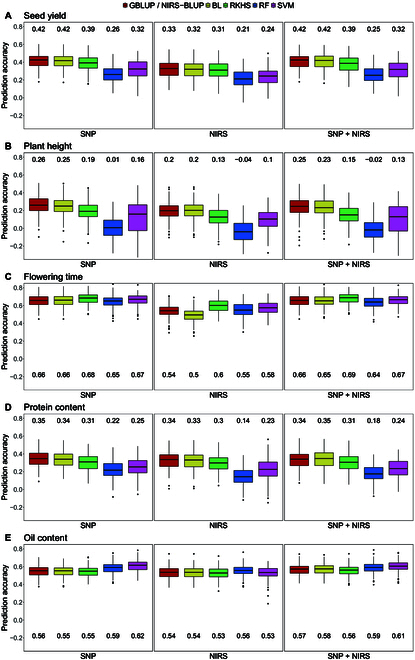
Comparison of prediction accuracy of GP based on SNP markers, PP based on NIRS data, and a combined approach based on both kinds of data with 5 different models (GBLUP/NIRS-BLUP, BL, RKHS, RF, and SVM) for seed yield (A), plant height (B), flowering time (C), protein content (D), and oil content (E) obtained by 200 cross-validation splits. Values above/underneath the boxplots represent median accuracies across all cross-validation runs. NIRS data was obtained from harvested seeds of the pollinators.

Figure 7 illustrates the outcome of this parental PP in comparison to GP and the combined approach integrating both data types. For seed yield (Fig. 7A), plant height (Fig. 7B), and flowering time (Fig. 7C), GP showed slight differences in accuracy compared to the prediction scenario within the hybrid generation presented in Fig. 2. This discrepancy arose from the exclusion of 9 genotypes in order to align results with the PP, as the NIRS data of the respective pollinators was missing. In contrast to the within-generation prediction, parental PP did not surpass GP for any trait and model. Nevertheless, the distinctions between the 2 approaches were generally slight. For seed yield, the accuracy of GP was, on average, 0.08 higher than that of PP. For plant height, GP outperformed models based on NIRS profiles by an average of 0.06. GP of flowering time already had outperformed the phenomic counterpart in the within-generation approach using NIRS data the hybrids themselves, and this trend remained consistent when parental NIRS profiles were utilized. However, 3 prediction models (RKHS, RF, and SVM) yielded higher accuracies for parental PP compared to within-generation PP. Oil and protein content were not predicted within the hybrid generation, as these values have been obtained through NIRS estimation, leading PP models to predict them with an accuracy of 1 or nearly 1. Parental PP for these 2 traits yielded accuracies almost identical to those of the GP for most models. The combined prediction approach resulted in accuracies very similar or even identical to those obtained by GP for all traits.

**Fig. 8.**
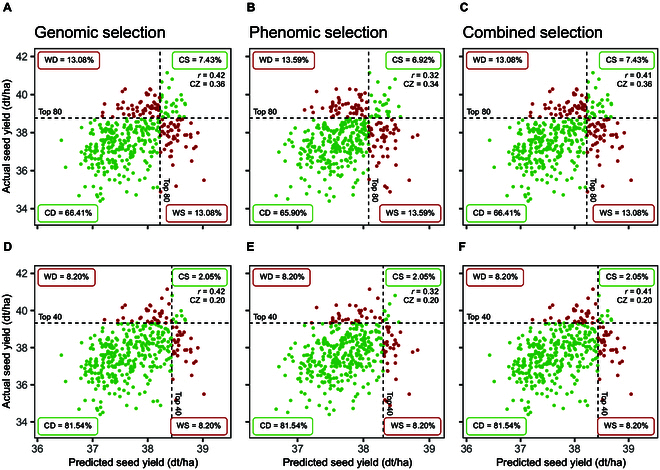
Comparison of selection accuracy of selecting the top 80 genotypes (A to C) or top 40 genotypes (D to F) with GS (A and D), PS (B and E), and combined selection (C and F) for seed yield based on the measured values (adjusted means) and predicted values (means of 200 cross-validations) with GBLUP/NIRS-BLUP. Genotypes that were in or out of the selected fraction based on both predicted and actual performance were classified as “correctly selected” (CS) or “correctly discarded” (CD), respectively. Genotypes that were in the selected fraction based on the prediction when in reality they performed worse, and vice versa, were classified as “wrongly selected” (WS) or “wrongly discarded” (WD), respectively. The respective percentage numbers indicate the corresponding faction sizes. Czekanowski coefficient of similarity (CZ) indicates the selection accuracy based on the predicted values. Pearson correlation coefficient (*r*) indicates the respective prediction accuracy. NIRS data was obtained from harvested seeds of the pollinators.

Comparing the accuracy of selecting genotypes with high seed yield, PS based on across-generation predictions with NIRS-BLUP performs nearly as well as GS based on predictions with GBLUP when selecting the top 80 genotypes (Fig. 8A and B). Here, GS and the combined selection resulted in a selection accuracy of 0.36, while PS achieved an accuracy of 0.34. When narrowing down to the top 40 genotypes, all approaches showed identical selection accuracy of 0.2, despite GP demonstrating a higher prediction accuracy than PP (Fig. 8D to F). The results for all other traits and prediction models are summarized in Tables S5 and S6. Overall, all 3 approaches demonstrate comparable levels of accuracy across all traits and models, and superiorities of individual approaches for certain traits are only very slight.

## Discussion

Since the publication of the work by Rincent et al. [11] suggesting PS as a cost-efficient alternative to GS, several studies have provided compelling evidence for its effectiveness in predicting important traits across various crops with high accuracy. Among the investigated crops are grapevine [22] and soybean [24], but the majority of the studies focus on cereals like maize [20,21], triticale [23], and wheat [17–19]. In this study, we aimed to evaluate the potential of PS for rapeseed breeding, with a specific focus on its applicability for hybrid breeding. Therefore, we predicted the performance of a hybrid population both within and across generations. Our analysis involved comparing the accuracy of these predictions as well as the precision in selecting the top-performing genotypes based on their predicted phenotypic values with GP and PP, respectively. Additionally, we assessed the ability of NIRS profiles derived from single locations to serve as a reference for predicting traits measured across multiple environments. We also investigated how population structure influences the accuracy of PP. Finally, we aim to provide a comprehensive overview of the utility and limitations of PS in the context of rapeseed breeding.

### PS can enhance early-stage selection efficiency in rapeseed breeding programs

The predictions within the hybrid generation confirmed that PP has the ability to outperform GP for specific traits (Fig. 2). While PP by far outperformed GP for the prediction of seed yield and plant height, it was not able to surpass the GP accuracy of flowering time. Zhu et al. [23] describe that the predictive ability of PS depends on the genetic architecture of the target trait. Applying PS to triticale, they found out that for traits with a complex genetic architecture, like grain yield, NIRS-based prediction outperformed GP, while for mono- or oligogenic traits, GP achieved much higher accuracies. The difference in accuracy between the 2 methods can be explained by the type of effects that are reflected by the different predictors. Models like GBLUP are based on the summation of the individual effects of multiple markers, each consisting of a single nucleotide. Hence, they mainly reflect additive genetic effects. On the other hand, as described by Zhu et al. [24], the reflectance values at each wavelength that is used by PS models is itself a phenotypic value that is influenced by some to many genes as well as the environment. Thus, PS models are able to include nonadditive genetic effects and environmental, as well as genotype by environment (G × E), effects. Therefore, the accuracy with which a trait can be predicted using either GP or PP reflects the extent to which that trait is influenced by additive genetic effects or other effects that can only be captured by spectral data, respectively. Accordingly, PS is superior to GS when complex traits are considered whose expression is strongly determined by environmental influences and G × E interactions, whereas GS performs better for less complex traits as it might be more efficient in capturing additive genetic effects.

For flowering time in rapeseed, multiple major effect quantitative trait loci (QTL) with high heritability, including key genes of the flowering pathway have been identified [64–67]. However, these major QTL mainly occur when using genetically very diverse germplasm sets comprising winter, semiwinter, and spring-type accessions, while they are most likely to be fixed in populations comprising only winter-type elite breeding accessions [68]. In contrast, the variation within one ecotype was shown to be environmentally dependent and controlled by multiple different genome regions with small effects [68,69]. In our study, flowering time obtained the highest prediction accuracies for GS and had the highest heritability of all considered traits. This indicates that flowering time was influenced mainly by genetic effects. Environmental factors, as well as G × E interactions, appeared to have less impact on flowering time than on seed yield and plant height. This would explain why PS failed to outperform GS here. The ability of PP to predict plant height more accurately than flowering time, even without a clear link between plant height and NIRS profiles of the seeds, might also be related to the fact that plant height has been demonstrated to have a higher correlation to seed yield, compared to flowering time [70].

Although these predictions were performed retrospectively, i.e., using NIRS data collected postharvest within the same generation as the traits being assessed, the results still show a high value for rapeseed breeding, particularly during early stages of a program. Here, breeders cultivate a large array of new genotypes in small, nonreplicated plots to screen for various qualitative traits. The limited size of these plots and the absence of multienvironmental replications often makes these early trials unsuitable for reliably determining the seed yield. In this context, PS can be a valuable tool enabling breeders to select not only based on qualitative traits but also already for complex traits such as seed yield. PS, therefore, has the potential to enhance early-stage selection efficiency in rapeseed breeding programs.

Consistent with the findings of previous studies [19,24,26,28], our results show that a combination of genomic and spectral data leads to higher prediction accuracies than using only one type of data. Following the hypothesis that GS can more precisely capture additive genetic effects, while PS is able to better account for nonadditive genetic, as well as environmental, and G × E interaction effects, it seems obvious that both methods complement each other when being combined. Hence, ongoing GS programs, where the genomic data is already available, can be improved by incorporating spectral data into the prediction models.

### NIRS-BLUP is a preferable model for implementing PS in breeding programs

For GP, many different statistical methods have been proposed and implemented, including linear regressions and nonlinear or machine learning models. Several studies compared multiple models in different datasets [3,71,72]. In summary, it can be stated that there is no single method that generally outperforms all other methods. Therefore, we decided to implement and compare 5 different models for all prediction scenarios carried out in this study.

While, as expected, none of the tested models outperformed all others consistently, there were still some noteworthy aspects. Regardless of the predictors used, the standard model GBLUP/NIRS-BLUP and the BL almost always led to the same prediction accuracies, aligning with the results of Zhu et al. [24]. The difference between these 2 models lies in the assumptions about the effects of the individual features. While GBLUP/NIRS-BLUP assumes that all SNP markers or wavelengths contribute equally to the phenotype, BL has the capability to apply specific shrinkage to each feature, potentially shrinking some feature effects to zero. This suggests that the differences in the effects of the individual features are negligible here, which could also explain why Zhu et al. [24] did not observe any improvement in prediction accuracy when selecting specific wavelengths.

RKHS performs on a comparable level to the standard model in many cases in this study. Interestingly, for the NIRS-based prediction of plant height within the same generation, it outperforms the NIRS-BLUP, while it is slightly less effective than the GBLUP when SNP markers are used (Fig. 2). However, for the parental prediction of plant height, RKHS also gets surpassed by NIRS-BLUP (Fig. 7). As RKHS is known to capture nonadditive genetic effects, including dominance and epistasis, this could be an indication that for some traits, there are complex, nonadditive relationships between the individual wavelength within the NIRS profiles. The fact that RKHS did not outperform the standard model when genomic data was used, nor when parental NIRS profiles were used as predictors, could suggest that these relationships are not of genetic nature but are shaped by environmental influences. When it comes to predicting the flowering time, the opposite trend is observed. Here, RKHS performs slightly better than GBLUP for GP and outperforms NIRS-BLUP for parental PP but results in a worse accuracy when using NIRS data within the same generation. This could indicate that here genetic nonadditive effects are captured by the model, which appear to be suppressed by environmental influences when predicting within the same generation. Therefore, the accuracy of RKHS in PP seems to depend not only on the genetic architecture of the trait but also on the trait-specific genotype–environment interaction.

The 2 tested machine learning models performed at a level comparable to the other models for flowering time but resulted in notably lower accuracies for seed yield and plant height when using SNP marker as predictors. This is contrary to many studies describing that RF and SVM have the ability to outperform linear GP models [73–75]. Conversely, the results are in agreement with the findings of Ogutu et al. [76], Waldmann [77], and Weber et al. [78]. Interestingly, both machine learning models perform much better when NIRS data is used as predictors. For PP of seed yield, both models reach the same level of accuracy as the other models, and for plant height, RF even yields the best prediction accuracies. For the combined approach, RF performs best for the prediction of both seed yield and plant height, and SVM reaches a level of accuracies that is comparable to the other models. A possible explanation for this could lie in the different information content of the 2 distinct predictors. The GP models are based on the number of the reference allele at each biallelic marker. Hence, there are only 3 different states per marker, as the reference allele can occur twice, once, or not at all. The models for PP in contrast are based on reflectance values at each wavelength within the NIRS profile. These values span a broad, continuous range and, therefore, provide numerous levels of Information. RF is a decision tree-based model, where the formation of such decision trees is predicated on splitting the data multiple times based on a determined threshold within a specific feature. It is conceivable that the model can define these thresholds more efficiently due to the higher information content within the individual features of the NIRS data. Nonetheless, the other models also seem to benefit from this higher information content, so the superiority of RF is only slight, if any. Additionally, the use of machine learning models involves more effort as the hyperparameter must be tuned for each run.

In conclusion, the basic model NIRS-BLUP shows sufficient competitiveness across different prediction scenarios, making it a preferable choice for the implementation of PS in breeding programs, especially when limited time precludes the testing and comparison of multiple models. Nonetheless, continued research into machine learning models for PS is warranted, particularly considering their promising capacity for effectively integrating various data types.

### NIRS data from single representative locations can be used for PS

To simplify the integration of PP into breeding programs, it would be advantageous to utilize NIRS profiles sourced from a single reference location. This approach reduces data input complexity and eliminates the need for adjustments of the reflectance values at each wavelength to account for environmental effects in each profile. Therefore, we assessed the suitability of each available location to serve as this reference location for obtaining NIRS profiles to predict traits across all locations.

As depicted in Fig. 4, our findings indicate that it is possible to achieve prediction accuracies that are comparable to, or even slightly surpassing, those using aggregated NIRS profiles from all locations. These results align with the findings of Zhu et al. [24]. Notably, certain locations consistently exhibited higher prediction accuracies, while others consistently demonstrated lower accuracies across all tested prediction models and traits. This indicates that some locations may be better suited to serve as reference location for providing NIRS profiles for PS than others, raising the question of how to choose the most suitable location.

Previous studies showed that NIRS profiles obtained from plants grown under drought stress seem to be better suited to predict traits measured under well-watered conditions than vice versa [11,20]. The authors hypothesize that the higher phenotypic differentiation in the stressed environment may lead to a more accurate training of the models and hence increase prediction accuracy. However, in these studies, the stressed and well-watered field trials were at the same location. Therefore, it remains to be determined how to choose the best among all the locations available to the breeder.

Examining the observed levels of seed yield (Table S1), it becomes clear that MOO, which consistently gave relatively high accuracies, displayed remarkable seed yield stability, with only an average difference of 1.91 dt/ha between 2020 and 2021. In contrast, ROS, the location with consistently lower accuracies across all models and traits, exhibited the greatest variation in seed yield between the 2 years, with a substantial difference of 23.36 dt/ha. Therefore, seed yield stability could serve as a potential indicator of a location’s suitability to act as a reference location for training PS models.

Another location that consistently yielded higher accuracies was RHH. This location demonstrated the second most stable seed yield across the 2 years, and notably, its average seed yield closely aligned with the overall average across all locations in both years. In contrast, HOH, which deviated the most from the average seed yield, proved least suitable for the prediction of seed yield. This observation suggests that locations most representative for the entire set of locations i.e., showing a level of seed yield that closely approximates the overall average, might be best suited for obtaining NIRS profiles for PS.

To comprehensively address the question of how to select the optimal location for obtaining NIRS profiles to predict various traits, further research is needed. This should include a broader range of locations and more extensive data collection over a longer period. Additionally, gathering and evaluating detailed environmental data from these locations would be beneficial in determining the most favorable environmental conditions for a potential reference location.

### PS is suitable for integrating genetically diverse breeding material

Population or family structure can considerably influence the accuracy of GS. A low degree of relatedness between the training and test set, such as the absence of full siblings from the training set within the test set, can lead to low prediction accuracies, and the use of unrelated families can even lead to accuracies close to zero or negative [78–82]. When the test set consists of a family not represented in the training set, the prediction of their breeding value relies solely on the Mendelian sampling term, rather than the parental average component. Hence, the accuracy of that prediction greatly depends on the diversity and relatedness of the training set to the predicted genotypes. High diversity ensures coverage of a broad range of haplotype combinations, and close relatedness maintains stable marker-QTL associations [83].

Our results indicate that, analogous to GP, PP demonstrates variable accuracies when different subpopulations form the test set (Fig. 6). This reflects the ability of NIRS profiles to capture genomic diversity. On the other hand, the prediction accuracies of PP often notably exceed those from GP, which may be caused by the capacity of NIRS profiles to also capture environmental effects. Additionally, for plant height and seed yield, accuracies of PP obtained by familywise cross-validation often exceeded the median accuracies of randomly cross-validated PP. In contrast, familywise cross-validated GP more often resulted in accuracies falling below the median accuracies of randomly cross-validated GP (Fig. S1). Consequently, the median prediction accuracies from familywise cross-validation align more closely with those from random cross-validation in PP than in GP, where the disparity between familywise and random cross-validation tends to be greater (Fig. S1). This indicates that PP seems to be more robust to family structure than GP, which aligns with the observations of Zhu et al. [24] and Weiß et al. [21]. Given that the subpopulation of the test set was exposed to the same environment as the other subpopulations forming the training set, the PP models likely benefited from capturing the influence of this shared environment. Therefore, the advantage of PP arises from its capacity to utilize environmental, or G × E interaction effects captured by NIRS profiles, rather than relying fully on predicting the Mendelian sampling term of the breeding value. This indicates that PS is particularly advantageous for the incorporation of diverse and less related new germplasm to expand the genetic basis of a breeding program. Again, this shows that PS is a promising tool, especially for early phases of breeding programs, where the integration of novel genetic diversity is essential.

### PS has potential to be applied for hybrid breeding

One objective of this study was to assess the potential of applying PS to hybrid breeding. Therefore, we used NIRS profiles obtained from the pollinator lines, grown in alongside the hybrids at one location, to predict the adjusted performance of the hybrids. Since the hybrids were obtained by crossing the pollinators with 2 different testers, we included the respective tester as a fixed factor in the prediction models. In contrast to the predictions within the hybrid generation, the results here indicate that PP can partially reach levels comparable to GP but does not outperform them for any of the considered traits. Nevertheless, when simulating the selection of the top-performing hybrids based on predicted phenotypic values, PS performs on the same levels of selection accuracy as GS. Further research is needed to determine the relationship between prediction accuracy, defined by the Pearson correlation coefficient comparing predicted and actual performance, and the selection accuracy, defined by the Czekanowski coefficient of similarity comparing selections based on predicted versus actual performance. Additionally, there is a need to identify the conditions under which different models exhibit varying levels of prediction accuracy while obtaining similar levels of selection accuracy. Ultimately, it should be explored if certain scenarios exist where a model may demonstrate lower prediction accuracy but higher selection accuracy compared to another model. In such cases, it warrants consideration whether incorporating the Czekanowski coefficient alongside the widely applied Pearson correlation could offer a more comprehensive evaluation of different prediction methods.

The choice of a prediction model for breeders to select the most promising candidates within their breeding material is influenced by a multitude of situational factors, making it a rather subjective decision. In addition to comparing prediction accuracies, determined through cross-validation within the training set, cost-effectiveness is also a crucial consideration. Taking into account that NIRS data is much easier to obtain than genotypic data, our results demonstrate that even though PP yields lower prediction accuracies, PS has potential to be preferred over GS for application in hybrid rapeseed breeding.

However, the hybrids investigated here were obtained by test crosses with 2 different testers, which allowed us to base the prediction models on the NIRS profiles of the pollinators. It remains to be evaluated how PS performs when applied to factorial mating designs where NIRS data obtained from both parents are used, and both GCA and specific combining ability effects are considered. Recent studies have demonstrated that sparse factorial designs are superior to test-cross designs for the formation of training sets used in GS [38,84,85] However, a shift from test crosses to more factorial mating requires performing and testing a higher number of crosses, which drastically increases the workload. This particularly applies to autogamous and partially autogamous plants like rapeseed, where crossings necessitate the development of male-sterile maternal lines and fertility restorers, which can be costly and time-consuming [39]. This underscores the importance of precisely predicting the hybrid performance and GCA of the parents to minimize the number of crosses carried out and tested. As with selection within generations, PS could be an alternative to GS that is cheaper, faster, and easier to apply.

### Conclusion

We explored the potential of implementing PS for hybrid rapeseed breeding programs. Our results demonstrate its value in selecting superior genotypes, particularly in the early stages of breeding programs, due to its ability to accurately predict complex traits within the hybrid generation, even with NIRS data from single reference locations. When choosing such a location, our findings suggest that those most representative of the target region should be preferred. In addition to a superior prediction accuracy compared to GP, we were able to confirm a lower susceptibility of PP to population structure. This underlines the high potential of PS for incorporating diverse and less related novel germplasm to expand the genetic basis of a breeding program. Our results support the hypothesis that PP models benefit from their ability to capture environmental and G × E interaction effects, in addition to genotypic effects. Interestingly, this advantage became evident even though we decided to use adjusted NIRS profiles for the predictions, aiming to enhance the detection of genotypic effects. This suggests that employing models incorporating multiple environmental-specific NIRS-based covariance matrices could further refine the precision in capturing G × E interactions, as observed in wheat by Robert et al. [19]. As a choice of prediction model, we recommend breeders to use the NIRS-BLUP, since it demonstrated sufficient competitiveness across various prediction scenarios without the need for time-consuming tuning of hyperparameters. While further research focusing on factorial crossing designs is needed regarding the selection of parental lines for hybrid production, our study already indicates that PP across generations is feasible, and selecting the best hybrids based on parental NIRS data is competitive to GS. Given the substantially lower costs and labor associated with PS, our study suggests that this approach represents a viable alternative to GS for rapeseed breeding. NIRS is typically measured across a broad spectrum, spanning at least several hundred wavelengths, to assess the content or composition of multiple metabolites. Thus, this data could be readily utilized to enhance or even replace GS programs. However, future research could explore the extent to which the number of wavelengths can be reduced while still maintaining sufficient prediction accuracy.

## Data Availability

For the underlying datasets and codes, please contact the corresponding author at Lennard.ehrig@agrar.uni-giessen.de.
