# Supplementary material for: Phenomic Selection for Hybrid Rapeseed Breeding
Source: Plant Phenomics. 2024 Jul 24;6:0215. doi: 10.34133/plantphenomics.0215 (PMC11268845; doi:10.34133/plantphenomics.0215)
Supplement: Supplementary 1 — Fig. S1 Tables S1 to S6 [file plantphenomics.0215.f1.zip › Table S1.docx]

Table S1: Descriptive statistics of the hybrid population for each trait (Seed yield, Flowering time, Plant height, Oil content, Protein content). min = minimum, max = maximum, µ = mean, sd = standard deviation, h² = heritability.

| Location | Year | Trait | | | | | | | | | | | | | | | | | | | | | | | | |
| --- | --- | --- | --- | --- | --- | --- | --- | --- | --- | --- | --- | --- | --- | --- | --- | --- | --- | --- | --- | --- | --- | --- | --- | --- | --- | --- |
|  |  | Seed yield [dt/ha] | | | | | Flowering time  [days after Jan. 1^st^] | | | | | Plant height [cm] | | | | | Oil content [%] | | | | | Protein content [%] | | | | |
|  |  | *min* | *max* | *µ* | *sd* | *h²* | *min* | *max* | *µ* | *sd* | *h²* | *min* | *max* | *µ* | *sd* | *h²* | *min* | *max* | *µ* | *sd* | *h²* | *min* | *max* | *µ* | *sd* | *h²* |
| HOH | 2020 | 38.08 | 68.08 | 54.67 | 5.33 | ­– | 100 | 105 | 101.81 | 1.16 | – | 110 | 177 | 141.66 | 10.82 | – | 48.55 | 53.30 | 50.95 | 0.79 | – | 13.20 | 17.55 | 15.33 | 0.72 | – |
|  | 2021 | 32.56 | 59.79 | 45.56 | 4.20 | – | 117 | 125 | 118.74 | 0.94 | – | 131.3 | 192.6 | 163.63 | 10.65 | – | 47.20 | 53.30 | 50.61 | 1.00 | – | 14.05 | 19.15 | 16.05 | 0.89 | – |
| LAU | 2020 | 19.27 | 43.03 | 33.35 | 3.54 | – | 97 | 105 | 101.05 | 1.37 | – | – | – | – | – | – | 47.75 | 51.20 | 49.33 | 0.62 | – | 15.80 | 18.90 | 17.31 | 0.61 | – |
|  | 2021 | 15.32 | 36.47 | 27.57 | 3.31 | – | 107 | 114 | 110.51 | 1.32 | – | – | – | – | – | – | 47.40 | 52.20 | 50.19 | 0.77 | – | 15.25 | 19.25 | 16.92 | 0.66 | – |
| MOO | 2020 | 27.45 | 55.39 | 40.14 | 5.68 | – | 106 | 111 | 108.24 | 0.78 | – | 72 | 122 | 101.22 | 8.46 | – | 48.75 | 53.60 | 51.46 | 0.80 | – | 13.55 | 17.85 | 15.21 | 0.63 | – |
|  | 2021 | 32.03 | 54.06 | 42.05 | 3.88 | – | 119 | 123 | 120.58 | 1.04 | – | 108 | 154 | 123.95 | 6.78 | – | 47.55 | 53.75 | 51.49 | 1.03 | – | 13.60 | 18.05 | 15.25 | 0.88 | – |
| RHH | 2020 | 18.92 | 50.90 | 37.94 | 5.49 | – | – | – | – | – | – | – | – | – | – | – | 49.15 | 53.75 | 51.89 | 0.80 | – | 12.60 | 16.40 | 14.13 | 0.70 | – |
|  | 2021 | 25.21 | 46.16 | 33.82 | 2.78 | – | 112 | 118 | 114.41 | 1.55 | – | 125 | 150 | 137.58 | 4.18 | – | 49.80 | 53.20 | 51.62 | 0.64 | – | 13.25 | 16.70 | 14.85 | 0.57 | – |
| ROS | 2020 | 34.16 | 58.01 | 44.86 | 3.50 | – | 100 | 103 | 101.99 | 0.68 | – | 120 | 165 | 141.24 | 8.09 | – | 48.45 | 53.60 | 51.84 | 0.71 | – | 12.25 | 17.50 | 14.16 | 0.60 | – |
|  | 2021 | 16.64 | 32.75 | 21.50 | 2.49 | – | 90 | 111 | 108.69 | 1.19 | – | 125 | 175 | 144.53 | 8.76 | – | 46.35 | 51.15 | 49.14 | 0.66 | – | 14.75 | 18.00 | 16.40 | 0.52 | – |
| Total | 2020 | 18.92 | 68.08 | 42.84 | 8.65 | – | 97 | 111 | 103.29 | 3.08 | – | 72 | 177 | 127.85 | 21.13 | – | 47.75 | 53.75 | 51.22 | 1.13 | – | 12.25 | 18.90 | 15.08 | 1.24 | – |
|  | 2021 | 15.32 | 59.79 | 34.09 | 9.52 | – | 90 | 125 | 114.59 | 4.73 | – | 108 | 192.6 | 142.42 | 16.37 | – | 46.35 | 53.75 | 50.62 | 1.24 | – | 13.25 | 19.25 | 15.88 | 1.04 | – |
|  | **Total** | **15.32** | **68.08** | **38.29** | **10.11** | **0.49** | **90** | **125** | **109.60** | **6.94** | **0.63** | **72** | **192.6** | **136.23** | **19.90** | **0.47** | **46.35** | **53.75** | **50.91** | **1.22** | **0.60** | **12.25** | **19.25** | **15.49** | **1.21** | **0.50** |
