# Supplementary material for: Phenomic Selection for Hybrid Rapeseed Breeding
Source: Plant Phenomics. 2024 Jul 24;6:0215. doi: 10.34133/plantphenomics.0215 (PMC11268845; doi:10.34133/plantphenomics.0215)
Supplement: Supplementary 1 — Fig. S1 Tables S1 to S6 [file plantphenomics.0215.f1.zip › Table S2.docx]

Table S2: Fraction sizes of the classification of genotypes based on the accuracy of selecting the top 80 genotypes for genomic selection (GS), phenomic selection (PS) and combined selection (CS) based on the measured values (adjusted means) and predicted values (means of 200 cross-validations). Genotypes that were in or out of the selected fraction based on both predicted and actual performance were classified as “correctly selected” or “correctly discarded”, respectively. Genotypes that were in the selected fraction based on the prediction when in reality they performed worse, and vice versa, were classified as “wrongly selected” or “wrongly discarded”, respectively. NIRS data was obtained from harvested seeds of the hybrids. Bold numbers indicate the best performance per trait and model.

| Model | Fraction size [%] for selecting the top 80 genotypes | | | | | | | | | | | |
| --- | --- | --- | --- | --- | --- | --- | --- | --- | --- | --- | --- | --- |
|  | Correctly discarded | | | Correctly selected | | | Wrongly discarded | | | Wrongly selected | | |
|  | *GS* | *PS* | *CS* | *GS* | *PS* | *CS* | *GS* | *PS* | *CS* | *GS* | *PS* | *CS* |
| **Seed yield:** |  |  |  |  |  |  |  |  |  |  |  |  |
| GBLUP/NIRS-BLUP | 66.9 | 68.7 | **69.4** | 7.0 | 8.0 | **9.5** | 13.0 | 13.0 | 13.0 | 13.0 | 13.0 | 13.0 |
| RKHS | 66.7 | 68.2 | **69.7** | 6.8 | 8.3 | **9.8** | 13.3 | 13.3 | 13.3 | 13.3 | 13.3 | 13.3 |
| Bayes. LASSO | 66.7 | 68.7 | **69.9** | 6.8 | 8.8 | **10.0** | 13.3 | 13.3 | 13.3 | 13.3 | 13.3 | 13.3 |
| RF | 67.2 | 68.2 | **69.4** | 7.3 | 8.3 | **9.5** | 12.8 | 12.8 | 12.8 | 12.8 | 12.8 | 12.8 |
| SVM | 65.9 | 68.4 | **69.4** | 6.0 | 8.5 | **9.5** | 14.0 | 14.0 | 14.0 | 14.0 | 14.0 | 14.0 |
| **Plant height:** |  |  |  |  |  |  |  |  |  |  |  |  |
| GBLUP/NIRS-BLUP | 62.9 | **68.4** | 68.2 | 3.0 | **8.5** | 8.3 | 17.0 | 11.5 | 11.8 | 17.0 | 11.5 | 11.8 |
| RKHS | 62.9 | **67.4** | **67.4** | 3.0 | **7.5** | **7.5** | 17.0 | 12.5 | 12.5 | 17.0 | 12.5 | 12.5 |
| Bayes. LASSO | 63.7 | **68.4** | 67.2 | 3.8 | **8.5** | 7.3 | 16.3 | 11.5 | 12.8 | 16.3 | 11.5 | 12.8 |
| RF | 62.7 | 67.4 | **68.4** | 2.8 | 7.5 | **8.5** | 17.3 | 12.5 | 11.5 | 17.3 | 12.5 | 11.5 |
| SVM | 62.4 | 67.9 | **68.4** | 2.5 | 8.0 | **8.5** | 17.5 | 12.0 | 11.5 | 17.5 | 12.0 | 11.5 |
| **Flowering time:** |  |  |  |  |  |  |  |  |  |  |  |  |
| GBLUP/NIRS-BLUP | **72.2** | 69.2 | 71.7 | **12.3** | 9.3 | 11.8 | 7.8 | 10.8 | 8.3 | 7.8 | 10.8 | 8.3 |
| RKHS | **72.9** | 67.9 | 72.4 | **13.0** | 8.0 | 12.5 | 7.0 | 12.0 | 7.5 | 7.0 | 12.0 | 7.5 |
| Bayes. LASSO | **72.4** | 68.9 | 72.2 | **12.5** | 9.0 | 12.3 | 7.5 | 11.0 | 7.8 | 7.5 | 11.0 | 7.8 |
| RF | **71.4** | 68.4 | **71.4** | **11.5** | 8.5 | **11.5** | 8.5 | 11.5 | 8.5 | 8.5 | 11.5 | 8.5 |
| SVM | 72.9 | 68.2 | **73.4** | 13.0 | 8.3 | **13.5** | 7.0 | 11.8 | 6.5 | 7.0 | 11.8 | 6.5 |
