# Supplementary material for: Phenomic Selection for Hybrid Rapeseed Breeding
Source: Plant Phenomics. 2024 Jul 24;6:0215. doi: 10.34133/plantphenomics.0215 (PMC11268845; doi:10.34133/plantphenomics.0215)
Supplement: Supplementary 1 — Fig. S1 Tables S1 to S6 [file plantphenomics.0215.f1.zip › Table S4.docx]

Table S4: Prediction accuracies of genomic prediction based on SNP markers, phenomic prediction based on NIRS data and a combined approach based on both kinds of data for predicting the performance of one subfamily (P1 – P5) when trained on the remaining four subfamilies with 5 different models (GBLUP/NIRS-BLUP, BL, RKHS, RF and SVM) for seed yield, plant height and flowering time. NIRS data was obtained within the hybrid generation from *harvested* seeds. Md = median.

| Model | Prediction accuracy | | | | | | | | | | | | | | | | | |
| --- | --- | --- | --- | --- | --- | --- | --- | --- | --- | --- | --- | --- | --- | --- | --- | --- | --- | --- |
|  | SNP | | | | | | NIRS | | | | | | SNP + NIRS | | | | | |
|  | *P1* | *P2* | *P3* | *P4* | *P5* | *Md* | *P1* | *P2* | *P3* | *P4* | *P5* | *Md* | *P1* | *P2* | *P3* | *P4* | *P5* | *Md* |
| **Seed yield:** |  |  |  |  |  |  |  |  |  |  |  |  |  |  |  |  |  |  |
| GBLUP/NIRS-BLUP | 0.19 | 0.11 | -0.06 | 0.22 | 0.40 | **0.19** | 0.61 | 0.68 | -0.01 | -0.03 | 0.38 | **0.38** | 0.66 | 0.66 | -0.02 | 0.09 | 0.44 | **0.44** |
| Bayes. LASSO | 0.21 | 0.09 | -0.05 | 0.24 | 0.42 | **0.21** | 0.65 | 0.68 | -0.02 | -0.02 | 0.38 | **0.38** | 0.69 | 0.62 | -0.01 | 0.16 | 0.45 | **0.45** |
| RKHS | 0.18 | 0.14 | -0.05 | 0.27 | 0.44 | **0.18** | 0.61 | 0.60 | -0.07 | -0.08 | 0.39 | **0.39** | 0.64 | 0.65 | -0.06 | 0.08 | 0.40 | **0.40** |
| RF | 0.05 | 0.07 | 0.09 | -0.15 | 0.11 | **0.07** | 0.63 | 0.58 | -0.02 | 0.01 | 0.35 | **0.35** | 0.72 | 0.63 | -0.04 | 0.14 | 0.35 | **0.35** |
| SVM | 0.21 | 0.08 | 0.02 | -0.09 | 0.23 | **0.08** | 0.47 | 0.40 | -0.01 | 0.00 | 0.35 | **0.35** | 0.67 | 0.61 | 0.00 | 0.13 | 0.40 | **0.40** |
| **Plant height:** |  |  |  |  |  |  |  |  |  |  |  |  |  |  |  |  |  |  |
| GBLUP/NIRS-BLUP | -0.02 | -0.30 | 0.04 | -0.09 | -0.40 | **-0.09** | 0.60 | 0.63 | -0.19 | 0.05 | 0.40 | **0.40** | 0.63 | 0.65 | -0.17 | 0.07 | 0.49 | **0.49** |
| Bayes. LASSO | -0.03 | -0.21 | 0.02 | -0.05 | -0.34 | **-0.05** | 0.62 | 0.63 | -0.19 | 0.05 | 0.41 | **0.41** | 0.65 | 0.70 | -0.18 | 0.09 | 0.49 | **0.49** |
| RKHS | -0.03 | -0.28 | 0.06 | -0.12 | -0.42 | **-0.12** | 0.68 | 0.71 | -0.11 | 0.10 | 0.34 | **0.34** | 0.67 | 0.74 | -0.09 | 0.11 | 0.42 | **0.42** |
| RF | 0.06 | 0.02 | 0.04 | 0.03 | 0.03 | **0.03** | 0.67 | 0.58 | -0.11 | 0.13 | 0.44 | **0.44** | 0.71 | 0.63 | -0.01 | 0.20 | 0.49 | **0.49** |
| SVM | 0.03 | -0.11 | 0.20 | 0.21 | -0.04 | **0.03** | 0.66 | 0.63 | -0.20 | 0.11 | 0.26 | **0.26** | 0.68 | 0.66 | -0.14 | 0.16 | 0.54 | **0.54** |
| **Flowering time:** |  |  |  |  |  |  |  |  |  |  |  |  |  |  |  |  |  |  |
| GBLUP/NIRS-BLUP | 0.46 | -0.06 | 0.23 | 0.26 | 0.46 | **0.26** | 0.32 | 0.28 | 0.22 | 0.48 | 0.37 | **0.32** | 0.46 | -0.06 | 0.23 | 0.40 | 0.39 | **0.39** |
| Bayes. LASSO | 0.46 | -0.04 | 0.27 | 0.31 | 0.48 | **0.31** | 0.32 | 0.26 | 0.23 | 0.47 | 0.36 | **0.32** | 0.46 | 0.03 | 0.25 | 0.45 | 0.46 | **0.45** |
| RKHS | 0.44 | 0.00 | 0.36 | 0.46 | 0.46 | **0.44** | 0.30 | 0.15 | 0.24 | 0.35 | 0.26 | **0.26** | 0.47 | 0.09 | 0.35 | 0.51 | 0.43 | **0.43** |
| RF | 0.32 | 0.00 | 0.17 | 0.25 | 0.32 | **0.25** | 0.21 | 0.14 | 0.26 | 0.18 | -0.14 | **0.18** | 0.29 | 0.09 | 0.27 | 0.25 | 0.26 | **0.26** |
| SVM | 0.51 | 0.08 | 0.29 | 0.41 | 0.49 | **0.41** | 0.22 | 0.21 | 0.18 | 0.01 | 0.10 | **0.18** | 0.46 | 0.18 | 0.32 | 0.52 | 0.48 | **0.46** |
