# Supplementary figures and images for: Phenomic Selection for Hybrid Rapeseed Breeding
Source: Plant Phenomics. 2024 Jul 24;6:0215. doi: 10.34133/plantphenomics.0215 (PMC11268845; doi:10.34133/plantphenomics.0215)

—●— random CV —●— familywise CV

**(a) Seed yield**

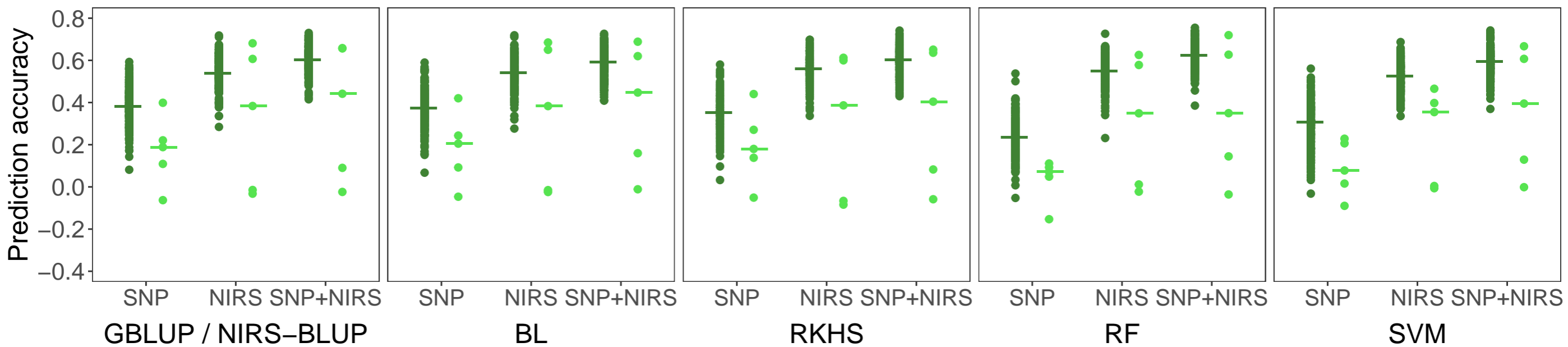

**(b) Plant height**

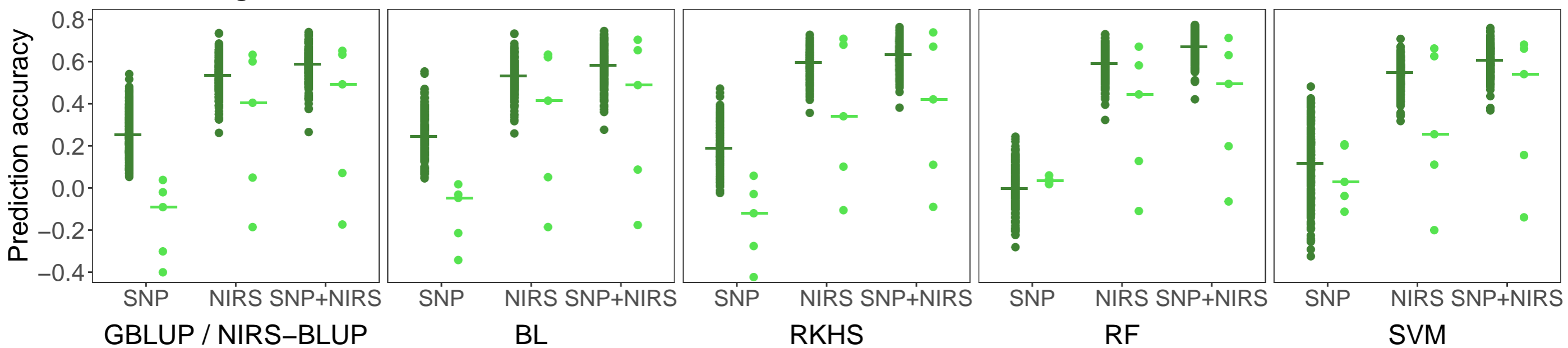

**(c) Flowering time**

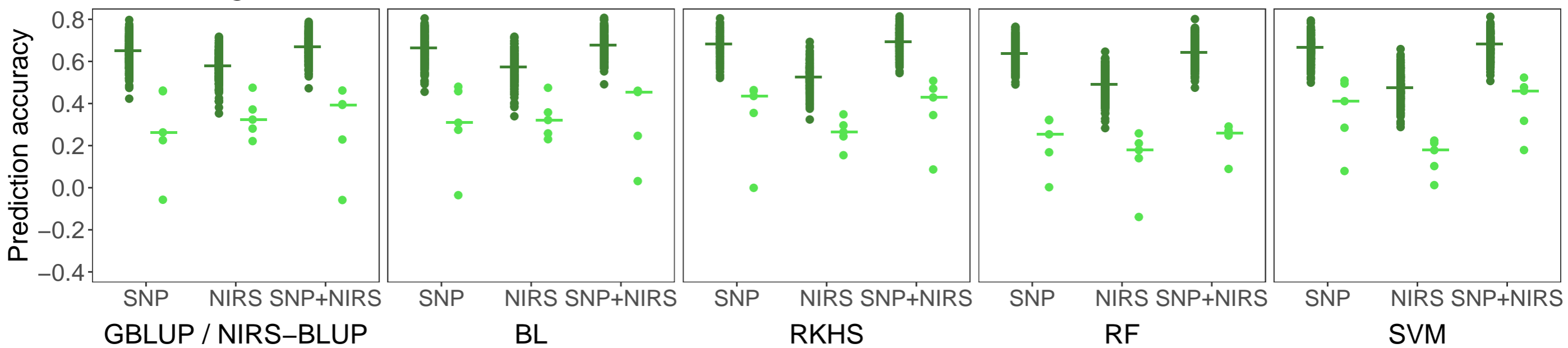

Supplement: Supplementary 1 — Fig. S1 Tables S1 to S6 [file plantphenomics.0215.f1.zip › FigureS1.pdf]
